# Longitudinal changes in insulin sensitivity, insulin secretion, beta cell function and glucose effectiveness during development of non-diabetic hyperglycemia in a Japanese population

**DOI:** 10.1186/2193-1801-3-252

**Published:** 2014-05-20

**Authors:** Toru Aizawa, Keishi Yamauchi, Masayuki Yamada

**Affiliations:** Diabetes Center, Aizawa Hospital, 2-5-1 Honjo, Matsumoto, Japan; Clinical Research Department, Kissei Pharmaceutical, Tokyo, Japan

**Keywords:** Non-diabetic hyperglycemia, Insulin sensitivity, Insulin secretion, Beta cell function, Glucose effectiveness

## Abstract

Since there had been no previous studies of alterations in insulin sensitivity, glucose-stimulated insulin secretion, beta cell function and glucose effectiveness during the development of non-diabetic hyperglycemia in Asian populations, we conducted a longitudinal study of such changes in 244 Japanese adults with normal glucose tolerance (median BMI 23.3 kg/m^2^ and age 51 yrs). The median follow-up period was 3.3 yrs. One hundred and eighty-two subjects maintained normal glucose tolerance (nonprogressors). After excluding the 3 subjects who progressed to diabetes, we analyzed the 59 who developed non-diabetic hyperglycemia (progressors), of which 31 progressed to impaired fasting glucose and 28 to impaired glucose tolerance. Whole body insulin sensitivity was estimated by ISI_Matsuda_, glucose-stimulated insulin secretion by [δIRI_0-30_/δPG_0-30_] and Stumvoll indices, hepatic insulin sensitivity by quantitative insulin sensitivity check index (QUICKI) and 1/fasting IRI, beta cell function by oral disposition index (DI_O_) ([δIRI_0-30_/δPG_0-30_]∙[ISI_Matsuda_]), and glucose effectiveness by an OGTT-derived index (SgI_O_). ISI_Matsuda_ (*p* <0.05), [δIRI_0-30_/δPG_0-30_], DI_O_ and SgI_O_ (both *p* <0.01), but not QUICKI, 1/fasting IRI, or Stumvoll-1st and -2nd phases, were lower in the progressors at baseline. This group was also characterized by the following: 1) ISI_Matsuda_, DI_O_ and SgI_O_ were reduced by 34%, 32% and 11%, respectively (all *p* <0.01); 2) QUICKI and 1/fasting IRI diminished by 21% and 5%, respectively (both *p* <0.01); and 3) no significant changes were found in [δIRI_0-30_/δPG_0-30_], Stumvoll-1st and -2nd phases or BMI during the follow-up. In the nonprogressors, no indices changed significantly during the follow-up. Our study concluded that during the transition from normal glucose tolerance to non-diabetic hyperglycemia in this non-obese population, whole body insulin sensitivity, hepatic insulin sensitivity, beta cell function, and glucose effectiveness were all attenuated, but no significant changes in glucose-stimulated insulin secretion occurred. Also of note is the fact that the transition took place without any accompanying increase in BMI.

**Electronic supplementary material:**

The online version of this article (doi:10.1186/2193-1801-3-252) contains supplementary material, which is available to authorized users.

## Background

In obese subjects with normal glucose tolerance (NGT), a lowering of insulin sensitivity (SI) during the development of non-diabetic hyperglycemia (NDH) has commonly been observed (Faerch et al. 2009: Ferrannini et al. 2011; Weyer et al. 1999). The picture with regard to glucose-stimulated insulin secretion (GSIS), however, has not been entirely consistent. In one previous study, GSIS *increased* significantly in the nonprogressors but *decreased* in the progressors (Weyer et al. 1999), whereas other investigators found that, while the indices of beta cell function decreased, the total insulin output over the 2 h of the oral glucose tolerance test increased in the progressors (Ferrannini et al. 2011). A third study reported no significant increase in GSIS in either the nonprogressors or progressors (Faerch et al. 2009).

In Japan, subjects in the general population are largely non-obese. Diabetes without overt obesity is common (Matsuba et al. 2012), and it is possible that the natural course of the worsening of glucose metabolism in the Japanese is different from that in obese populations (Faerch et al. 2009: Ferrannini et al. 2011; Weyer et al. 1999). Cross-sectional studies have shown that SI and GSIS were significantly attenuated in Japanese subjects with minute elevations of plasma glucose (PG) within the normal range, and the attenuation was progressively greater with increasing levels of PG across NDH and diabetes mellitus (DM) (Sato et al. 2002; Oka et al. 2012). However, there have been no reports of longitudinal alterations in SI, GSIS or beta cell function (BCF) during the progression from NGT to NDH in Japanese subjects, or in any other non-obese population.

In a previous study, we found that the attenuation of whole body SI and GSIS at baseline in Japanese subjects with NGT was significantly related to a worsening of the glucose metabolism (Oka et al. 2014a). Our next step was to attempt to clarify the longitudinal changes in SI, GSIS and BCF during the progression from NGT to NDH. Because the attenuation of glucose effectiveness (Sg) has been identified as an important element in the worsening of the glucose metabolism (Goldfine et al. 2003; Tonelli et al. 2005), we also examined the longitudinal changes in Sg by using an OGTT-derived index, SgI_O_ (Nagasaka et al. 2012).

## Results and discussion

The baseline characteristics for the health examinees with NGT whose immunoreactive insulin (IRI) was determined at the follow-up OGTT were similar to those whose IRI was not measured at the follow-up OGTT, except that the subjects in the former group were slightly younger and had slightly lower 2hIRI values. Importantly, the incidence rate of NDH did not differ significantly between the two groups (Additional file 1: Table S1). It was the examinees whose IRI had been measured upon follow-up (*n* = 244) who became the subjects of the present study.

At the end of the follow-up, 182 participants remained in the NGT category and were classified as nonprogressors. The rest had progressed to NDH or DM. Those who had progressed to impaired fasting glucose (IFG) (*n* = 31) and impaired glucose tolerance (IGT) (*n* = 28) were collectively classified as progressors (*n* = 59), and the data from both the nonprogressors and progressors were analyzed. The subjects who progressed to DM (*n* = 3) were excluded from the analysis.

The characteristics of the 241 subjects analyzed in this study are shown in Table 1. Male gender and a family history of diabetes were significantly more frequent in the progressors than in the nonprogressors, and the changes in fasting plasma glucose (FPG) and 2hPG in male subjects were significantly greater compared to those in female subjects (*p* = 0.04 for FPG and *p* <0.01 for 2hPG). At baseline, BMI, FPG, 2hPG and 2hIRI were significantly higher in the progressors than in the nonprogressors. On the other hand, ISI_Matsuda_, insulinogenic index (δIRI_0-30_/δPG_0-30_), the product of insulinogenic index and ISI_Matsuda_ (DI_O_II), the product of Stumvoll-1 and ISI_Matsuda_ (DI_O_ST-1) and SgI_O_ were significantly lower in the progressors than in the nonprogressors at baseline. During the follow-up, none of the indices in the nonprogressors changed significantly. In the progressors, ISI_Matsuda_, the quantitative insulin sensitivity check index (QUICKI) and 1/fasting IRI (1/FIRI) decreased by 34%, 5% and 21%, respectively (all *p* <0.01). δIRI_0-30_/δPG_0-30_ increased while Stumvoll-1 and -2 decreased but in neither case significantly. The progressors also experienced attenuation of DI_O_II, DI_O_ST-1 and SgI_O_ by 32% (*p* <0.05), 32% (*p* <0.01) and 11% (*p* <0.01), respectively, during the follow-up, with minimal, insignificant decreases in BMI. Changes in ISI_Matsuda_ and progression to NDH were significantly correlated after adjustment for age, gender, family history of diabetes, baseline BMI, follow-up period and change in BMI in the entire population (*β* = -0.15, *p* = 0.02).

**Table 1:** **Characteristics of the participants analyzed in this study**

Variables		All	Subgroup			
			Non-progressors		Progressors	
*n*	241		182		59	
Age, yr	51(46–57)		50(45–57)		52(47–57)	
Men/women	163/78		113/69		50/9§	
Family Hx of diabetes, +/-	32/209		19/163		13/46‡	
	Baseline	Follow-up	Baseline	Follow-up	Baseline	Follow-up
BMI, kg/m^2^	23.3(21.3–25.3)	23.2(21.3–25.4)	22.9(21.0–24.9)	23.0(20.9–25.0)	24.4(22.6–24.4)	24.2(22.8–26.0)
FPG, mg/dL	93(89–96)	94(90–97)†	92(89–95)	92(89–95)	95(91–97)	101(94–105)†
2hPG, mg/dL	104(93–116)	106(92–119)†	102(91–113)	103(90–114)	114(103–123)	134(109–155)†
FIRI, μU/mL	3.4(2.6–4.8)	3.6(2.8–5.1)	3.3(2.6–4.5)	3.4(2.7–4.6)	3.7(2.6–5.3)	4.4(3.6–5.8)†
2hIRI, μU/mL	19.3(12.3–29.6)	21.6(14.0–35.8)†	18.5(10.8–27.2)	19.5(13.4–32.2)	25.1(16.6–35.0)	31.9(19.9–49.5)†
ISI_Matsuda_	12.8(9.3–18.8)	11.5(7.5–16.4)†	13.2(10.2–19.4)	12.4(9.3–17.3)	11.1(8.3–17.8)	7.3(5.3–11.0)†
QUICKI	0.400(0.378–0.421)	0.396(0.376–0.417)*	0.400(0.380–0.424)	0.401(0.381–0.422)	0.393(0.372–0.420)	0.377(0.363–0.391)†
1/FIRI	0.29(0.21–0.38)	0.28(0.20–0.36)	0.30(0.22–0.39)	0.29(0.22–0.37)	0.28(0.19–0.40)	0.23(0.17–0.28)†
Insulinogenic index	0.61(0.36–1.10)	0.63(0.40–1.03)	0.65(0.39–1.12)	0.69(0.45–1.12)	0.48(0.29–0.90)	0.52(0.29–0.91)
Stumvoll-1	740.1(529.4–952.5)	753.2(571.3–1033.0)	755.5(561.7–967.4)	776.8(619.5–1030.3)	694.8(504.2–864.5)	638.2(448.7–869.8)
Stumvoll-2	205.2(161.6–251.8)	209.5(174.1–263.2)	206.8(168.4–252.8)	211.0(182.2–267.8)	199.0(155.3–236.5)	188.8(146.0–237.1)
DI_O_	7.78(4.38–15.52)	6.89(3.48–12.06)	8.73(5.27–17.2)	8.25(5.18–15.23)	5.16(3.28–9.33)	3.24(2.09–5.72)†
SgIo	3.27(2.87–3.88)	3.28(2.73–3.76)	3.41(2.93–3.97)	3.43(2.94–3.93)	3.04(2.65–3.65)	2.70(2.19–3.36)†
Ratio						
ISI_Matsuda_	0.84(0.61–1.73)		0.96(0.64–1.31)		0.66(0.45–0.86)§	
QUICKI	1.00(0.94–1.10)		1.00(0.96–1.05)		0.95(0.90–1.01)§	
1/FIRI	0.97(0.71–1.79)		1.00(0.79–1.31)		0.79(0.58–1.06)§	
Insulinogenic index	0.99(0.66–1.59)		0.99(0.68–1.58)		1.01(0.63–1.86)	
Stumvoll-1	1.02(0.78–1.65)		1.03(0.80–1.31)		0.98(0.68–1.44)	
Stumvoll-2	1.03(0.83–1.50)		1.03(0.84–1.23)		1.02(0.78–1.31)	
DI_O_	0.88(0.45–1.61)		0.93(0.47–1.70)		0.68(0.37–1.27)‡	
SgIo	0.99(0.91–1.05)		1.01(0.95–1.06)		0.89(0.78–0.96)§	
Follow-up period, yr	3.3(2.2–4.1)		3.4(2.1–4.1)		3.1(2.2–4.7)	

Numerical data is the median (25–75 percentile). Progressors comprise those who progressed to impaired fasting glucose and impaired glucose tolerance. BMI, body mass index; FPG, fasting plasma glucose; 2hPG, PG 2-h after oral intake of 75 g glucose; FIRI, fasting immunoreactive insulin; 2hIRI, IRI after oral intake of 75 g glucose; ISI_Matsuda_, Matsuda insulin sensitivity index; QUICKI, quantitative insulin sensitivity check index; Insulinogenic index, δIRI_0-30_/δPG_0-30_; Stumvoll-1, Stumvoll index of first phase insulin secretion; Stumvoll-2, Stumvoll index of second phase insulin secretion; DI_O_, oral disposition index (product of Insulinogenic index and ISI_Matsuda_); SgI_O_, index of glucose effectiveness derived from OGTT. Ratio denotes [follow-up value/baseline value] of each variable in each subject. *and †, *p* <0.05 and <0.01, respectively, compared to the corresponding baseline values by Wilcoxon’s signed rank test; ‡ and §, *p* <0.05 and <0.01, respectively, compared to the corresponding values in nonprogressors by Fisher’s exact test or Wilcoxon’s signed rank test. See Text for detail.

When, at the end of the follow-up, a group-wise comparison was made between the nonprogressors and the progressors, the absolute values for BMI, FIRI, and 2hIRI were significantly higher, and ISI_Matsuda_, 1/FIRI, QUICKI, δIRI_0-30_/δPG_0-30_, Stumvoll-1 and -2, DI_O_II, DI_O_ST-1, and SgI_O_ significantly lower in the latter group (*p* <0.05 for Stumvoll-2 and <0.01 for other variables). Because baseline values differed significantly for some variables between the nonprogressors and the progressors, the degrees of change, i.e. the ratios, in the nonprogressors and the progressors were also compared by Mann-Whitney U test. FIRI and 2hIRI increased more significantly in the progressors than in the nonprogressors and ISI_Matsuda_, 1/FIRI, QUICKI, DI_O_II, DI_O_ST-1 and SgI_O_ were more significantly attenuated in the nonprogressors than in the progressors.

In this study, we aimed to clarify the longitudinal changes during the development of NDH in Japanese subjects, a non-obese population. The seminal findings were as follows. In the progressors, ISI_Matsuda_, QUICKI, 1/FIRI, DI_O_II, DI_O_ST-1 and SgI_O_ were attenuated during the follow-up, which was associated with insignificant changes in δIRI_0-30_/δPG_0-30_, Stumvoll-1 and Stumvol-2. In the nonprogressors, none of the variables showed significant changes during the follow-up. Overall, our data showed that an attenuation of hepatic and whole body SI, BCF and Sg, on top of already lowered whole body SI, early phase GSIS, BCF and Sg, took place during the transition from NGT to NDH in this population. Among the changes occurring during the development of NDH, the lowering of whole body SI and BCF was the most prominent, and the lowering of whole body SI and progression to NDH were independently correlated. Also of note is the fact that in the progressors, these changes occurred in the absence of weight gain.

There are both similarities and dissimilarities between our data and the data obtained in previous longitudinal studies which analyzed the natural course of the development of NDH in obese, WHO-defined NGT subjects (Faerch et al. 2009: Ferrannini et al. 2011; Weyer et al. 1999). A lowering of SI in the progressors during the development of NDH is a common finding in all studies (Faerch et al. 2009: Ferrannini et al. 2011; Weyer et al. 1999; current study). On the other hand, the GSIS data differ significantly (Faerch et al. 2009: Ferrannini et al. 2011; Weyer et al. 1999; current study). In a study of Pima Indians (Weyer et al. 1999), GSIS *increased* significantly with attenuation of SI in the nonprogressors but *decreased* in the progressors. In Europids, the indices of beta cell function, such as glucose sensitivity, decreased while total insulin output over the 2 h of the OGTT increased slightly in the progressors (Ferrannini et al. 2011). In another study (Faerch et al. 2009), GSIS did not increase significantly in either in the nonprogressors or the progressors. The study subjects were markedly (Weyer et al. 1999) or slightly (Faerch et al. 2009; Ferrannini et al. 2011) obese, and the participants had further gained weight during the follow-up in all studies (Faerch et al. 2009: Ferrannini et al. 2011; Weyer et al. 1999) except ours (current study). The ‘hyperinsulinemia’ reported in one study (Weyer et al. 1999) may partly have been a reflection of ‘hyperproinsulinemia’ caused by cross-reaction of IRI with proinsulin (Larsson and Ahrén 1999). As far as we are aware, there has been no longitudinal study in which Sg was evaluated during the development of NDH. The present study employed a recently reported index of Sg derived from OGTT (Nagasaka et al. 2012), and is the first documentation of a significant attenuation of Sg with progression from NGT to NDH.

In summary, the major differences between the obese subjects of previous studies and the non-obese subjects in our study were as follows. In the former, there were increases in GSIS in the nonprogressors (Weyer et al. 1999) and decreases in the progressors (Weyer et al. 1999). The indices of beta cell function decreased but total insulin output over the 2 h of the OGTT increased in the progressors (Ferrannini et al. 2011). Furthermore, there was further weight gain in both the nonprogressors and progressors in the obese subjects. In contrast, there were no changes either in GSIS or body weight not only in the nonprogressors but also in the progressors in the non-obese population of our study.

A significant lowering of whole body SI occurred in the progressors without weight gain. This suggested that some factor other than weight gain must have been responsible for the lowering of SI in the progressors. Hepatic steatosis (Oka et al. 2014b; Burgert et al. 2006), sarcopenia (Booth et al. 2012; Narici and Maffulli 2010) and glucose toxicity (Solomon et al. 2012; Del Prato et al. 1994) are all possible underlying mechanisms. The reasons for male preponderance among the progressors are unclear. However, this is compatible with the fact that NDH is more prevalent in men than in women in the general population (Matsuba et al. 2012; Cowie et al. 2009).

The study had its limitations. The sample size was small and the OGTT data consisted of indices of measurements. The follow-up period was not very long so that the eventual disposition of the participants was unknown. The number of subjects who developed NDH was not very large and therefore a subanalysis after separation of the progressors into IFG- and IGT-progressors was not feasible. It is possible that the indices of insulin secretion and insulin sensitivity may be different between IFG and IGT even in non-obese populations and the circumstances of the present study precluded the performance of a sub-group analysis. The indices of SI, GSIS, BCF and Sg were all functions of FPG and 2hPG by definition except for 1/FIRI. In particular, determining BCF by applying DI from OGTT is potentially problematic on account of autocorrelation since both 1st-phase insulin secretion and ISI_Matsuda_ employed the same parameters of fasting glucose and fasting insulin. Therefore, a degree of caution is called for when interpreting the relationship between alterations in the indices and changes in PG. Due to the study design, an unintentional selection bias cannot be entirely ruled out.

## Conclusions

Diminution of whole body and hepatic SI, BCF and Sg occurred during transition from NGT to NDH without significant changes in GSIS or body weight in this population. The unchanged GSIS might be an adaptation failure for decreased SI. This may be a common feature of non-obese populations in the early stage of worsening of the glucose metabolism.

## Methods

### Study sample

A retrospective observational study was conducted using a dataset provided by the Health Service Department of Hokuriku Central Hospital, where public school employees receive annual medical checkups. Out of an initial 604 consecutive subjects with NGT who received a subsequent 75 g OGTT (Oka et al. 2014a; Yamauchi et al. 2013), 244 whose IRI was measured both at the basal and subsequent OGTT before March 2012 (the median follow-up period being 3.3 yrs), were selected for further investigation. All of them were Japanese. Signed informed consent was obtained from all subjects, and the Hokuriku Central Hospital Review Board approved the study protocol.

We recommended measurement of IRI to the examinees receiving OGTT, and 244 took up this option, which was offered at an additional cost of approximately 10 euros. It is possible, therefore, that there was an unintentional socio-economic selection bias, i.e., that relatively wealthy people were preferentially recruited. Nevertheless, if there were any baseline phenotypic differences between those who accepted and those who declined our proposal regarding IRI measurement, these differences were neglible (see below).

### Measurement of plasma glucose and insulin, and diagnosis of glucose tolerance

PG was analyzed by the glucose oxidase method and IRI by the chemiluminescence method (ADVIA Centaur, Siemens Medical Solutions). The IRI assay does not cross-react with proinsulin (Marcovina et al. 2007). IRI values lower than the detection limit of the assay, 0.4 μU/mL (*n* = 8), were assumed to be a half of the assay limit, i.e., 0.2 μU/mL. The diagnosis of glucose tolerance category was made according to the 2003 ADA criteria (The Expert Committee on the Diagnosis and Classification of Diabetes Mellitus 2003).

### Calculations

ISI_Matsuda_ was calculated as an index of whole body SI using the fasting and 2 h blood samples: ISI_Matsuda_ = 10,000/[sqrt(FPG∙2hPG∙FIRI∙2hIRI)] (DeFronzo and Matsuda 2010) where FPG and 2hPG denote fasting PG and PG 2 h after 75 g oral glucose load, and FIRI and 2hIRI denote fasting and 2-h post load IRI, respectively. The quantitative insulin sensitivity check index (QUICKI) (Katz et al. 2000) and 1/FIRI (Hermans et al. 1999) were calculated as indices of primarily hepatic SI (Soonthornpun et al. 2003): QUICKI = 1/[log(FPG) + log(FIRI)]. For ISI_Matsuda_, QUICKI and 1/FIRI, the unit of PG and IRI was mg/dL and μU/mL, respectively (DeFronzo and Matsuda 2010; Katz et al. 2000; Hermans et al. 1999). It should be noted that 1/FIRI is not a function of PG values so that it is free from any autocorrelation with FPG and 2hPG. As an index of early phase GSIS, insulinogenic index (δIRI_0-30_/δPG_0-30_) during 75 g OGTT, was employed (Kosaka et al. 1974). As alternative indices of GSIS, Stumvoll 1st (Stumvoll-1) and 2nd phase (Stumvoll-2) indices, respectively, were also used: Stumvoll-1 = 1283 + 1.829∙IRI_30_ – 138.7∙PG_30_ + 3.772∙FIRI and Stumvoll-2 = 287 + 0.4164∙IRI_30_ – 26.07∙PG_30_ + 0.9226∙FIRI, in which the unit of IRI and PG was pmol/L and mmol/L, respectively (Stumvoll et al. 2000). BCF was estimated by oral disposition indices (DI_O_): a product of insulinogenic index and ISI_Matsuda_ (DIoII) (Nagasaka et al. 2012; Aizawa et al. 2012) and a product of Stumvoll-1 and ISI_Matsuda_ (DIoST-1) (Oka et al. 2014a; Yamauchi et al. 2013) were employed. As an index of Sg, SgI_O_ which is derived from OGTT (Nagasaka et al. 2012) was calculated. The degree of change for each variable was quantified by calculating [follow-up data/baseline data] ratio. Minus values for Stumvoll-1 and -2 were obtained in 4 and 1 subjects, respectively, and δIRI_0-30_/δPG_0-30_ could not be calculated in 13 subjects because the numerator or denominator was zero or minus, and the values for these subjects were assumed to be absent.

### Statistics

SPSS version 21.0 was used for statistical analysis. Due to non-normal distribution of the data, all numerical data were expressed as median (25th - 75th percentile). Mann-Whitney U test, Wilcoxon’s signed-rank test, Chi square test and partial correlation were used as needed, and *p* <0.05 was considered significant.

## Electronic supplementary material

Additional file 1: Table S1: Baseline characteristics of the NGT health examinees who received and not received IRI measurement at the follow-up OGTT. (DOCX 48 KB)

## Abbreviations

ADA: American Diabetes Association
BCF: Beta cell function
DIO: Oral disposition index
DIoII: A product of insulinogenic index and ISI_Matsuda_
DIoST-1: A product of Stumvoll 1st phase index and ISI_Matsuda_
DM: Diabetes mellitus
FIRI: Fasting immunoreactive insulin
FPG: Fasting plasma glucose
GSIS: Glucose stimulated insulin secretion
SG: Glucose effectiveness
IFG: Impaired fasting glucose
IGT: Impaired glucose tolerance
IRI: Immunoreactive insulin
SgIO: Index of glucose effectiveness derived from OGTT
NGT: Normal glucose tolerance
PG: Plasma glucose
SI: Insulin sensitivity
Stumvoll-1: Stumvoll 1st phase index
Stumvoll-2: Stumvoll 2nd phase index
2hPG: PG at 120 min with 75 g OGTT
2hIRI: IRI at 120 min with 75 g OGTT
QUICKI: Quantitative insulin sensitivity check index.
