# Supplementary material for: Longitudinal changes in insulin sensitivity, insulin secretion, beta cell function and glucose effectiveness during development of non-diabetic hyperglycemia in a Japanese population
Source: Springerplus. 2014 May 20;3:252. doi: 10.1186/2193-1801-3-252 (PMC4039663; doi:10.1186/2193-1801-3-252)
Supplement: Supplementary file 1 — Additional file 1: Table S1: Baseline characteristics of the NGT health examinees who received and not received IRI measurement at the follow-up OGTT. (DOCX 48 KB) [file 40064_2014_972_MOESM1_ESM.docx]

**Table S1.** Baseline characteristics of the NGT health examinees who received and not received IRI measurement at the follow-up OGTT

| Variables | Folow-up IRI measurement (+) | Follow-up IRI measurement (-) |
| --- | --- | --- |
| *N* | 244 | 360 |
| Age, yr | 51(46-57)† | 54(48-58) |
| Men/women | 165/79 | 236/124 |
| BMI, kg/m^2^ | 23.2(21.4-25.2) | 23.4(21.9-25.1) |
| FPG, mg/dL | 93(89-96) | 93(89-96) |
| 2hPG, mg/dL | 104(93-117) | 106(92-119) |
| FIRI, µU/mL | 3.4(2.6-4.8) | 3.7(2.8-5.1) |
| 2hIRI, µU/mL | 19.2(12.3-29.5)* | 21.5(14.3-32.3) |
| Follow-up period, yr | 3.2(2.2-4.1) | 3.9(2.6-4.7) |
| Status at the follow-up OGTT  NGT/NDH/DM | 182/59/3 | 308/50/2 |

Continuous data is the median (25^th^ - 75^th^ percentile). IRI, immunoreactive insulin; BMI, body mass index; FPG, fasting plasma glucose; 2hPG, PG 2-h after oral intake of 75 g glucose; FIRI, fasting IRI; 2hIRI, IRI 2-h after oral intake of 75 g glucose; NGT, normal glucose tolerance; NDH, non-diabetic hyperglycemia; DM, diabetes. Difference between the 2 groups was analyzed by Mann-Whitney U test for continuous variables and Chi square test for categorical variables. * and †, *p* <0.05 and <0.01, respectively. See Text for detail.
